# The specific absorption rate in different brain regions of rats exposed to electromagnetic plane waves

**DOI:** 10.1038/s41598-019-49719-4

**Published:** 2019-09-16

**Authors:** Hao-Yu Wang, Chun-Fang Li, Chao Yu, Ji Dong, Yong Zou, Bin-Bin Nie, Jia-Kai Li, Lin Ma, Rui-Yun Peng

**Affiliations:** 1 Beijing Institute of Radiation Medicine, Beijing, 100850 China; 2 First Medical Center of PLA General Hospital, Beijing, 100853 China; 3 Beijing Engineering Research Center of Radiographic Techniques and Equipment, Institute of High Energy Physics, Chinese Academy of Sciences, Beijing, 100049 China; 4 Hainan Hospital of PLA General Hospital, Sanya, 572013 Hainan China

**Keywords:** Biophysics, Biological physics

## Abstract

Accurate dosimetry of a specific brain region in rats exposed to an electromagnetic field (EMF) is essential for studies focusing on dose-effect relationship of the region. However, only dosimetry of whole brain or whole body were evaluated in most of previous studies. In this study, a numerical voxel rat model with 10 segmented brain regions was constructed. Then, the effects of frequency, incidence direction, and E-polarization direction of plane wave EMF on brain region averaged specific absorption rate (BRSAR) of rats were investigated. At last, the reliability of using whole-body averaged SAR (WBDSAR) and whole-brain averaged SAR (WBRSAR) as estimations of BRSAR were also evaluated. Our results demonstrated that the BRSAR depended on the frequency, incidence direction, and E-polarization direction of the EMF. Besides, the largest deviation could be up to 13.1 dB between BRSAR and WBDSAR and 9.59 dB between BRSAR and WBRSAR. The results suggested that to establish an accurate dose-effect relationship, the variance of the BRSAR induced by alteration of frequency, incidence direction, and E-polarization direction of EMF should be avoided or carefully evaluated. Furthermore, the use of WBDSAR and WBRSAR as estimations of BRSAR should be restricted to certain conditions such that the deviations are not too large.

## Introduction

Accompanied by the booming of wireless technology, electromagnetic field (EMF) exposure is now ubiquitous in everyday life. Safety concerns for public and occupational exposure have promoted extensive studies on biological effects induced by EMF in recent decades. Previous studies demonstrated that the brain is one of the most sensitive organs to EMF radiation. Under certain conditions, EMF exposure is considered to be associated with altered blood-brain barrier (BBB) permeability^1,2^, memory and learning function^3,4^, and physiological indexes^5^.

Studies on small animals, especially rats, are usually conducted to determine the EMF dose-effect relationship in the brain, which can then be translated to humans^6^. Accurate evaluation of power deposited in the brains of rats is essential for establishing the dose-effect relationship. Specific absorption rate (SAR) is one of the most well-known indexes implemented to quantify the power deposition in a biological subject, which is defined by the following formula^7^,

1 $${\rm{SAR}}=\frac{\sigma }{\rho }\cdot {|\overrightarrow{E}|}^{2},$$

where *σ* and *ρ* denote the conductivity and density of the tissue, respectively. $\overrightarrow{E}$ denotes the electric filed in the tissue. The local SAR for EMF radiation depends on many extrinsic and intrinsic parameters such as frequency, polarization, animal size, posture, etc.^8–10^.

Instead of whole brain dose-effect relationship, a number of studies focused on the EMF dose-effect relationship in specific brain regions such as the hippocampus^11^, striatum^12^, cerebral cortex^13^, and cerebellum^14^. Considering the non-uniformity of SAR distribution in the brain^15^, precise evaluation of EMF radiation dose delivery in target brain regions of rats is the precondition for accurate interpretations and validations of biological effects, especially when the investigation is based on detailed histopathology^16,17^. However, in most of these previous studies, only approximate whole-body/whole-brain averaged SAR (WBDSAR/WBRSAR) were implemented rather than the brain region averaged SAR (BRSAR). The use of WBDSAR/WBRSAR as substitutions of BRSAR may diminish the accuracy of dosimetry analysis. The reproducibility of the studies on biological effects induced by EMF relies on accurate dosimetry analysis which guarantees the same energy deposition in the subjects across different studies. If imprecise dosimetry analysis is implemented in a new study, the biological effects of previous studies may not be reproduced or a conflict result may be achieved.

In actuality, SAR distribution in rat brain is hard to obtain by direct measurement, especially for the *in vivo* experiments. Therefore, the numerical methods based on voxel rat models had been widely used to evaluate the SAR distribution. Realistic numerical voxel models can be constructed based on magnetic resonance imaging (MRI) or computer tomography (CT) data^6,18^. Based on voxel models, numerical methods such as finite difference time domain (FDTD) scheme can be performed to provide high resolution SAR distribution^7^.

Most of the voxel rat models have taken rat brain as a unified structure, although some of them considered substructures of rat brains such as white matter, grey matter, thalamus, and cerebellum^3,8,14,16^. The segmentation of rat brain voxel model can provide more accurate BRSAR which is essential especially for the studies focusing on biological effects induced by EMF on specific brain regions and their related functions (e.g. the hippocampus and memory). However, none of the previous voxel rat models have segmented the rat brain into detailed regions that are directly related to certain functions. As a matter of fact, there is no systemic study on the influence of plane wave EMF radiation with different configurations on SAR in different brain regions of rats.

The main purpose of this study is to investigate the effect of frequency, incidence direction and E-polarization direction of EMF on BRSAR in specific rat brain regions. Furthermore, the reliability of using WBDSAR and WBRSAR as estimations of BRSAR should also be evaluated. To accomplish these goals, a numerical voxel rat model with 10 segmented brain regions were constructed based on a digital rat brain atlas. Then, FDTD scheme were implemented to numerically assess the BRSAR value of each brain region under EMF exposure with 12 different configurations (three E-polarization directions, each of which had four incidence directions) at different frequencies ranging from 0.1 GHz to 2.45 GHz.

## Methods

### Rat model with 10 brain regions

**Figure 1:**
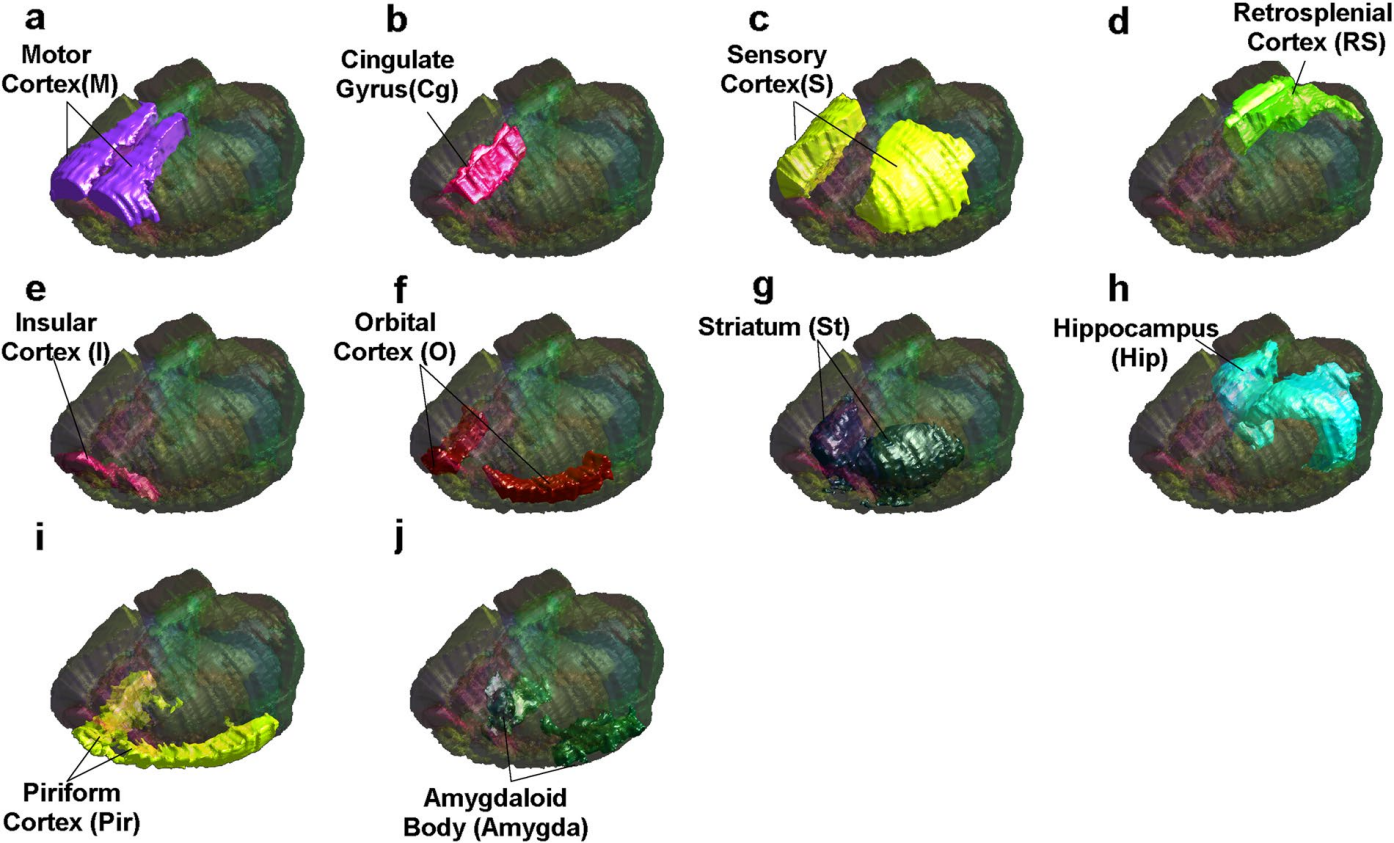
Numerical voxel model of rat brain with 10 segmented brain regions. (**a**–**j**) Demonstrate the relative locations of the motor cortex (M), cingulate gyrus (Cg), sensory cortex (S), retrosplenial cortex (RS), insular cortex (I), orbital cortex (O), striatum (St), hippocampus (Hip), piriform cortex (Pir), and amygdaloid body (Amygda), respectively.

The rat brain model constructed in our work was generated from a digital rat brain atlas offering detailed anatomical information based on the 5th edition rat brain stereotaxic coordinates built by Paxinos and Watson^19^. The construction of the digital rat brain atlas was described in details in ref.^20^. Based on the digital atlas (spatial resolution = 0.14 × 0.14 × 0.25 mm^3^, matrix size = 120 × 80 × 98), 3D digital rat brain model was subsequently generated using iSeg (ZMT AG, Zurich, Switzerland). Motor cortex (M), cingulate gyrus (Cg), sensory cortex (S), retrosplenial cortex (RS), insular cortex (I), orbital cortex (O), striatum (St), hippocampus (Hip), piriform cortex (Pir), and amygdaloid body (Amygda) were defined on the rat brain digital atlas in Paxinos and Watson space and saved as the rat brain model with 10 brain regions (Fig. 1). The rat brain model was then imported in Sim4life (version 3, Zurich Med Tech AG, Zurich, Switzerland), registered and substituted into the rat numerical voxel model with 51 segmented tissues.

### FDTD calculation

Detailed dosimetric calculations were performed using the FDTD method in Sim4life to evaluate the BRSAR in the 10 brain regions as well as the WBDSAR and WBRSAR. The weights and volumes of all segmented brain regions are listed in Supplementary Table 1S. Other specific parameters in the simulations were as follows:

1. Incident power density was 1 mW/cm^2^.
2. Grid size was set to 0.5 × 0.5 × 0.5 mm^3^ in segmented rat brain regions to guarantee convergent results and accurate SAR values in specific tissues^21^. For other tissues, the grid size was automatically assigned by the Sim4life at the refinement level of ‘fine’.
3. The dielectric properties (relative permittivity ${{\rm{\varepsilon }}}_{r}$ and conductivity $\sigma$) at 30 frequencies ranging from 0.1 GHz to 2.45 GHz were in accordance with IT’IS database Version 3.0^22^. The dielectric properties of brain tissue at all frequencies involved in this study are listed in Supplementary Table 2S. For tissues and organs that had not been listed, dielectric properties from tissues with similar anatomical compositions were assigned.
4. Definition of 12 configurations of plane wave EMF exposure are listed in Table 1. Incidence direction meant the direction of wave vector $\overrightarrow{k}$ and E-polarization direction meant the direction of electric field vector $\overrightarrow{E}$. Both of the incidence and E-polarization directions were denoted by two letters. DV denoted the direction from dorsal to ventral, VD denoted the direction from ventral to dorsal, AP denoted the direction from anterior to posterior, PA denoted the direction from posterior to anterior, LR denoted the direction from left to right, RL denoted the direction from right to left. The names of all configurations consisted of four letters. The first two letters indicated the incidence direction and the second two indicated the E-polarization direction.

**Table 1:** Definition of configurations of plane wave EMF exposures.

Configuration	Incidence direction^*^	E-Polarization direction^**^
DVAP	dorsal to ventral	anterior to posterior
VDAP	ventral to dorsal	anterior to posterior
LRAP	left to right	anterior to posterior
RLAP	right to left	anterior to posterior
APDV	anterior to posterior	dorsal to ventral
PADV	posterior to anterior	dorsal to ventral
LRDV	left to right	dorsal to ventral
RLDV	right to left	dorsal to ventral
APLR	anterior to posterior	left to right
PALR	posterior to anterior	left to right
DVLR	dorsal to ventral	left to right
VDLR	ventral to dorsal	left to right

^*^Incidence direction: the direction of wave vector $\overrightarrow{k}$.

^**^E-Polarization direction: the direction of electric field vector $\overrightarrow{E}$.
5. For the truncation of the computational region, the authors adopted a perfectly matched layer (PML) as the absorbing boundary.

All simulations were conducted on a workstation with the configurations as follows: CPU: Xeon E3-1225 3.2 GHz (Intel, Santa Clara, CA), memory: 16GB, and GPU: QUADRO K2200 (Nvidia, Santa, Clara, CA) with 2 GB memory.

### Data analysis

To investigate the influence of incidence direction on SAR value, the variations of BRSAR (*V*) among different incidence directions were calculated for each E-polarization direction, using the following formula,

2 $$V=\frac{BRSA{R}_{max}-BRSA{R}_{min}}{BRSA{R}_{mean}}\times 100 \% ,$$

where BRSAR_max_, BRSAR_min_, and BRSAR_mean_ denoted the maximum, minimum, and average BRSAR value of different configurations of the plane wave EMF radiation under the current investigation. For example, to investigate the influence of incidence direction of plane wave EMF on BRSAR, the incidence direction varied while the E-polarization direction was fixed. When the E-polarization was fixed to AP direction, four configurations (DVAP, VDAP, LRAP, and RLAP) of plane wave EMF were under investigation. The BRSAR_max_, BRSAR_min_, BRSAR_mean_ were the maximum, minimum, and average BRSAR values of these four configurations, respectively. Similarly, the variations of BRSAR among different E-polarization directions were also calculated for each incidence direction to assess the influence of E-polarization direction. Higher *V* suggested larger disparity of BRSAR, and vice versa.

To evaluate the reliability of using WBDSAR as an estimation of the BRSAR, the deviations between WBDSAR and BRSAR (*D*_WBDSAR_) at three selected frequencies (0.8 GHz, 0.9 GHz, and 2.45 GHz), at which the related bio-effect had been investigated extensively, were calculated using the following formula,

3 $${D}_{{\rm{WBDSAR}}}=|10\cdot {\log }_{10}\frac{BRSAR}{WBDSAR}|.$$

To evaluate the reliability of using WBRSAR as an estimation of BRSAR, the deviations between BRSAR and WBRSAR (*D*_WBRSAR_) were also calculated using Eq. (3) by replacing WBDSAR with WBRSAR. Higher *D*_WBDSAR_ (or *D*_WBRSAR_) implied less reliability of using WBDSAR (or WBRSAR) as an estimation of BRSAR, and vice versa.

## Results

### WBDSAR, WBRSAR, and BRSAR vs frequency

To investigate the influence of frequency, BRSAR as well as the WBDSAR and WBRSAR at different frequencies were calculated by numerical simulations using FDTD. The comparison of WBDSAR at different frequencies for 12 EMF exposure configurations is shown in Supplementary Fig. S1. In the range of 0.1~2.0 GHz, the WBDSAR of AP E-polarization configurations (DVAP, VDAP, RLAP, and LRAP) were always higher than other configurations. Moreover, whole-body resonance peaks could be observed at 0.45 GHz for AP E-polarization configurations. For configurations that the incidence directions were parallel to the axial direction of rat and the E-polarization directions were in radial directions of rat (APDV, PADV, APLR, PALR), two WBDSAR peaks could be observed for each case. The first peaks located at 0.5 GHz and the second ones located in the range of 1.1~1.2 GHz. For configurations that both the incidence and E-polarization directions were in radial directions of rat (LRDV, RLDV, DVLR, VDLR), the WBDSAR generally increased along with the increasing frequency and no obvious peaks were found.

Comparisons of WBRSAR at different frequencies for the configurations with fixed E-polarization directions are illustrated in Supplementary Fig. S2. For most AP E-polarization configurations (DVAP, LRAP, and RLAP), there were two WBRSAR peaks for each case. The first peaks located in the range of 0.4~0.45 GHz and the second ones located in 1~1.2 GHz depending on different configurations. But for the case of VDAP, there was only one obvious peak at 0.5 GHz. For DV E-polarization configurations, when the incidence directions were parallel to the axial direction of rat (APDV and PADV), there existed two WBRSAR peaks for each case. The first peaks located in the range of 1~1.2 GHz and the second ones located at 1.8 GHz. When the configurations were LRDV and RLDV, single WBRSAR peaks were found in the range of 1.3~1.4 GHz. For LR E-polarization configurations, the WBRSAR values were generally lower than the other two E-polarization directions. There were two WBRSAR peaks for APLR, PALR, and DVLR. The first peaks located in the range of 1~1.1 GHz and the second ones located in 2~2.2 GHz. There was only one obvious peak at 1.6 GHz for VDLR.

**Figure 2:**
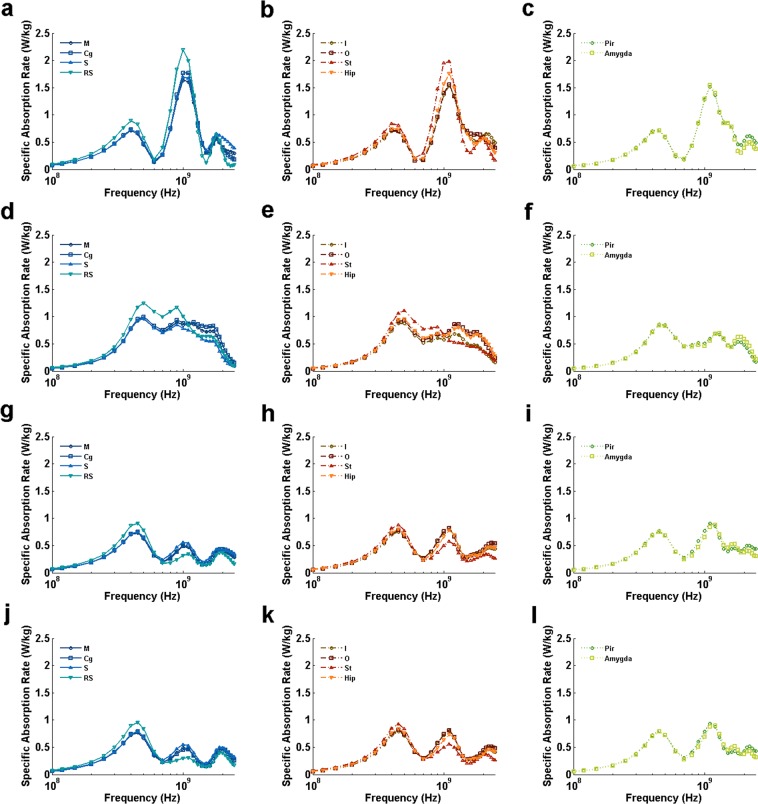
BRSAR vs frequency in different rat brain regions for AP E-polarization configurations. (**a**–**l)** Illustrate the BRSAR at different frequencies in semilog coordinates when the configurations of EMF are DVAP, VDAP, LRAP, and RLAP, respectively. M: motor cortex; Cg: cingulate gyrus; S: sensory cortex; RS: retrosplenial cortex; I: insular cortex; O: orbital cortex; St: striatum; Hip: hippocampus; Pir: piriform cortex; Amygda: amygdaloid body.

**Figure 3:**
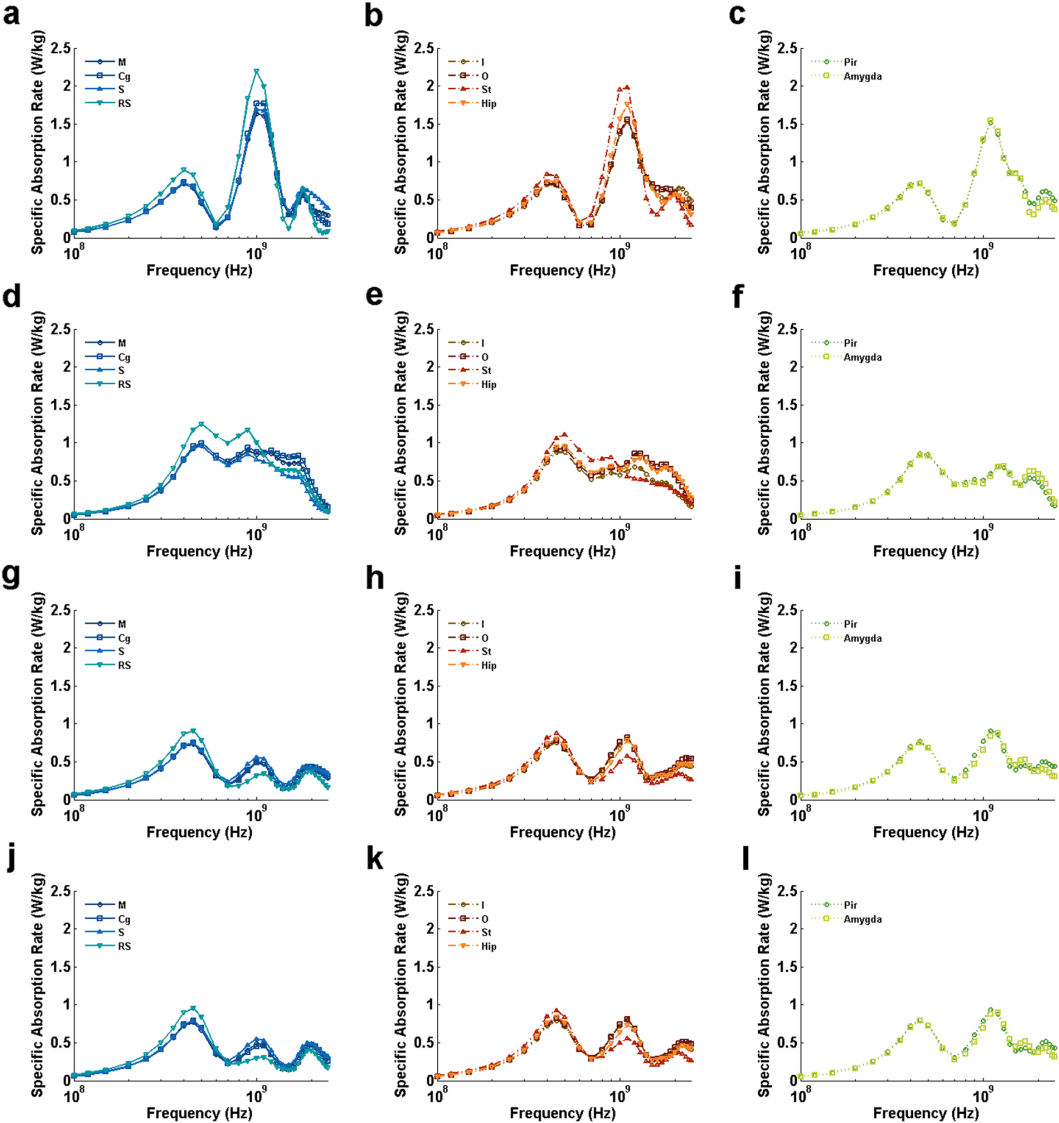
BRSAR vs frequency in different rat brain regions for DV E-polarization configurations. (**a**–**l**) Illustrate the BRSAR at different frequencies in semilog coordinates when the configurations of EMF are APDV, PADV, LRDV, and RLDV, respectively. M: motor cortex; Cg: cingulate gyrus; S: sensory cortex; RS: retrosplenial cortex; I: insular cortex; O: orbital cortex; St: striatum; Hip: hippocampus; Pir: piriform cortex; Amygda: amygdaloid body.

**Figure 4:**
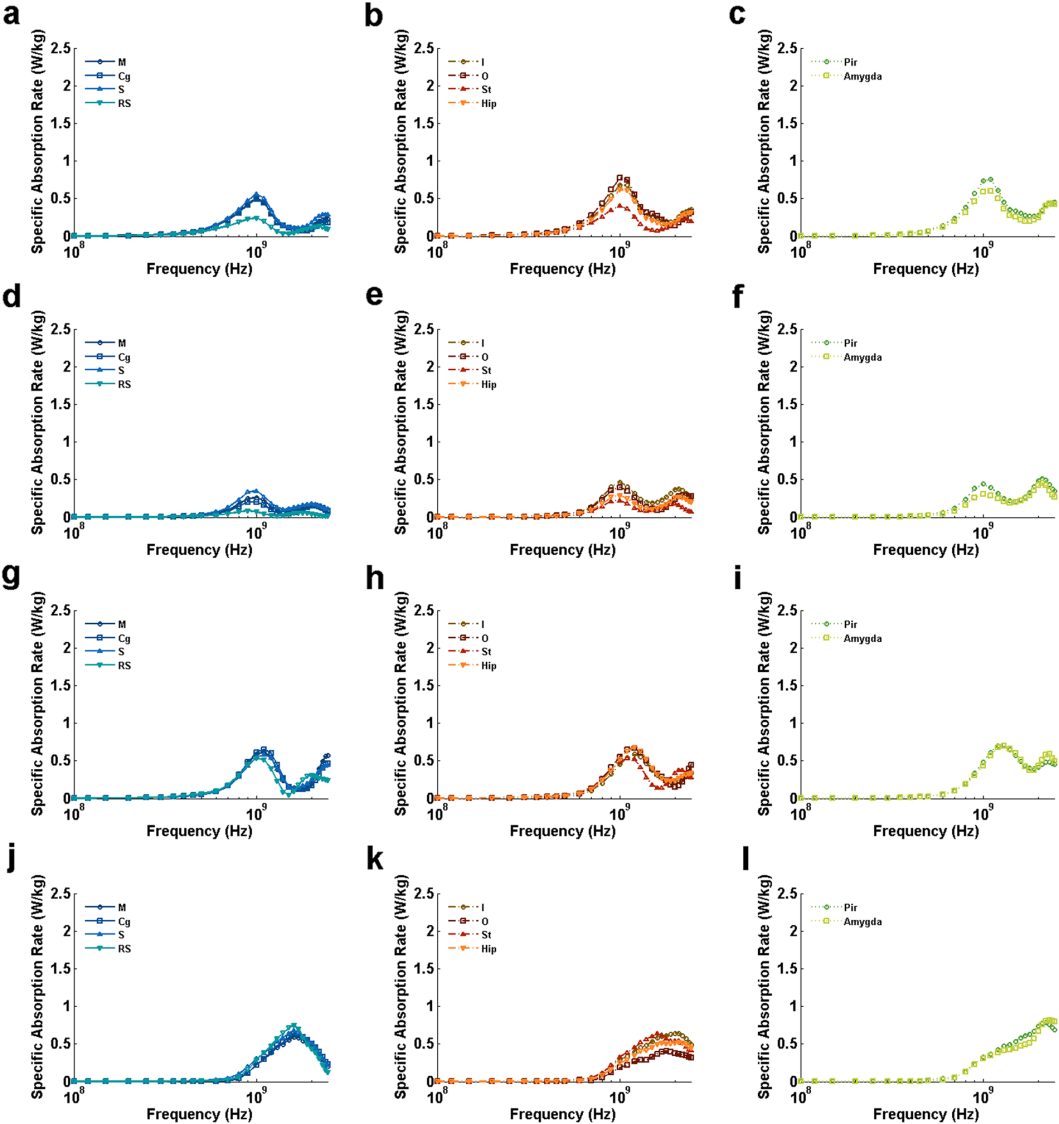
BRSAR vs frequency in different rat brain regions for LR E-polarization configurations. (**a**–**l**) Illustrate the BRSAR at different frequencies in semilog coordinates when the configurations of EMF are APLR, PALR, DVLR, and VDLR, respectively. M: motor cortex; Cg: cingulate gyrus; S: sensory cortex; RS: retrosplenial cortex; I: insular cortex; O: orbital cortex; St: striatum; Hip: hippocampus; Pir: piriform cortex; Amygda: amygdaloid body.

BRSAR at different frequencies are shown in Figs 2, 3 and 4. For all configurations with AP E-polarization (Fig. 2), the first BRSAR peaks could be observed around whole-body resonance frequency (0.45~0.5 GHz) in all brain regions. The second BRSAR peaks could be found in the range of 0.9~1.2 GHz. The third BRSAR peaks under DVAP exposure were ranging from 1.8 GHz to 2.2 GHz depending on different brain regions (Fig. 2a–c). For VDAP, the third BRSAR peaks located in the range of 1.2~1.3 GHz in M, Cg, I, O, Hip, Pir, and Amygda (Fig. 2d–f). For LRAP and RLAP, the third BRSAR peaks were in the range of 1.7~2.3 GHz (Fig. 2g–l). The fourth BRSAR peaks under VDAP exposure located in the range of 1.7~1.9 GHz in M, Cg, I, O, Hip, Pir, and Amygda (Fig. 2d–f). For LRAP and RLAP, the fourth peaks could be found at 2.1~2.2 GHz in ventral brain regions such as Pir and Amygda. It was also obvious that the BRSAR values under LRAP and RLAP exposure were similar to each other due to the symmetric property of rats.

For DV E-polarization (Fig. 3), two BRSAR peaks could be found in most brain regions when the incidence directions were parallel to the axial direction of rat (APDV and PADV, see Fig. 3a–f, respectively). Exceptions were found in some dorsal brain regions (M for APDV, see Fig. 3a; M, Cg, S and RS for PADV, see Fig. 3d) and orbital cortex (for PADV, see Fig. 3e) that only single peaks could be found at frequencies ranging from 1.0 GHz to 1.1 GHz. When the incidence directions were from one side of rat to the other (LRDV and RLDV, see Fig. 3g–l, respectively), single peaks can be observed at frequencies ranging from 1.2 GHz to 1.7 GHz.

For LR E-polarization (Fig. 4), obvious BRSAR peaks could be found at frequencies ranging from 0.9 GHz to 1.1 GHz in most brain regions under APLR (Fig. 4a–c) and DVLR (Fig. 4g–i) exposures. There existed a second BRSAR peak for some brain regions (RS and St for APLR, see Fig. 4a,b, respectively; St, Pir, and Amygda for DVLR, see Fig. 4h,i, respectively). For PALR (Fig. 4d–f), two BRSAR peaks could be found. The first peaks located in the range of 1.0~1.3 GHz and the second ones located in 2.0~2.3 GHz. For VDLR (Fig. 4j–l), only single BRSAR peaks were found at 1.6~2.3 GHz depending on the brain region.

### Effect of incidence direction of EMF on BRSAR

**Figure 5:**
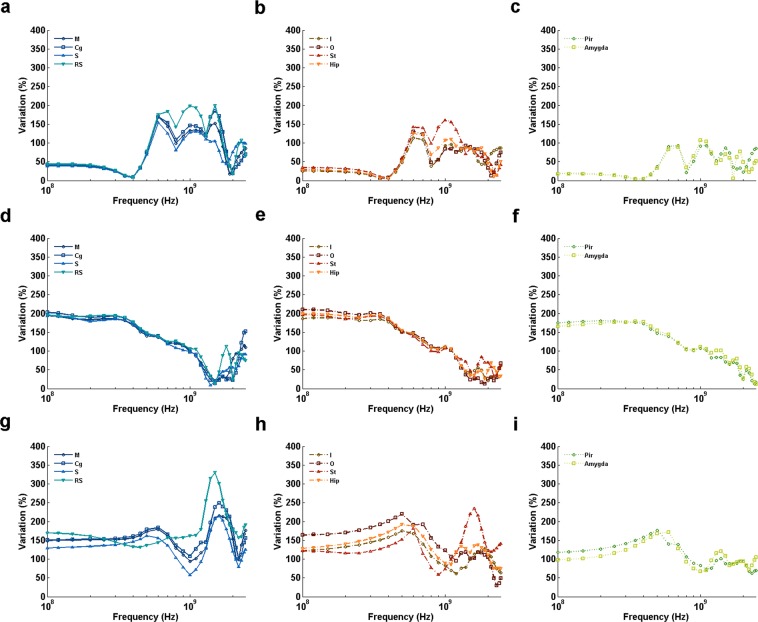
Variation of BRSAR in different brain regions when the direction of E-polarization is fixed and the incidence direction varies. (**a**–**i**) Show the variation of SAR values in brain regions at different frequencies in semilog coordinates when the polarizations are AP, DV, and LR, respectively. M: motor cortex; Cg: cingulate gyrus; S: sensory cortex; RS: retrosplenial cortex; I: insular cortex; O: orbital cortex; St: striatum; Hip: hippocampus; Pir: piriform cortex; Amygda: amygdaloid body.

To investigate the effect of incidence direction, the variations of BRSAR among different incidence directions were calculated for each E-polarization direction in the range of 0.1~2.45 GHz. For AP E-polarization (Fig. 5a–c), the BRSAR variations gradually decreased in the range of 0.1~0.4 GHz and then increased quickly to a higher level at 0.6 GHz. The BRSAR variations fluctuated around a relatively high level after 0.6 GHz and decreased under 50% around 1.7~2.3 GHz. The ventral brain regions such as Pir and Amygda (Fig. 5c) had relatively lower variation than other regions. For DV E-polarization (Fig. 5d–f), generally speaking, the BRSAR variations decreased as the frequency increased and became lower than 50% around 1.4~2 GHz. For LR E-polarization (Fig. 5g–i), the BRSAR variations of most brain regions first gradually increased in the range of 0.1~0.6 GHz, then decreased to a valley around 0.9~1.2 GHz. In RS region, the BRSAR variations fluctuated around 150% in the range of 0.1~1.1 GHz. In some brain regions (M, Cg, S, RS, I, St, Pir, and Amygda), BRSAR variation peaks could be found in the range of 1.4~1.9 GHz.

### Effect of E-polarization direction of EMF plane wave on BRSAR

**Figure 6:**
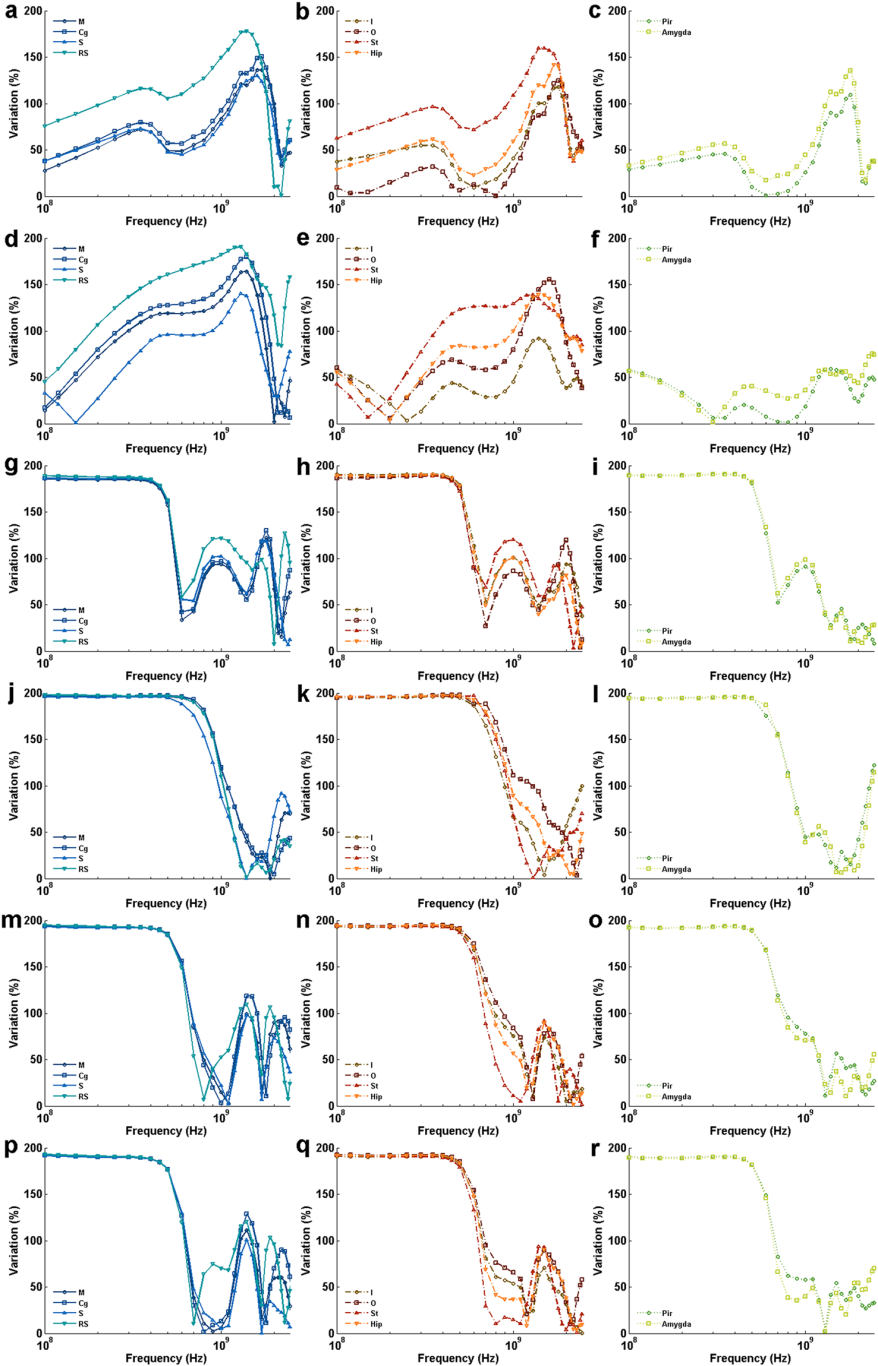
Variation of BRSAR when the incidence directions are fixed and the E-polarization direction varies. (**a**–**r**) Show the variation of BRSAR at different frequencies in semilog coordinates when the incidence directions are AP, PA, DV, VD, LR, and RL, respectively. M: motor cortex; Cg: cingulate gyrus; S: sensory cortex; RS: retrosplenial cortex; I: insular cortex; O: orbital cortex; St: striatum; Hip: hippocampus; Pir: piriform cortex; Amygda: amygdaloid body.

To evaluate the effect of E-polarization direction, the variations of BRSAR among different E-polarization directions were calculated for each incidence direction in the range of 0.1~2.45 GHz. For AP incidence, the variations of BRSAR had two peaks in all brain regions (Fig. 6a–c). The first peaks located at 0.35 GHz and the second ones located in the range of 1.4~1.8 GHz. The highest BRSAR variation was at 1.4 GHz in the RS region (~178%) for AP incidence configurations. For PA incidence, the BRSAR variations depended on different brain regions (Fig. 6d–f). In most of the dorsal brain regions (M, Cg, and S), the variations of BRSAR generally increased as the frequency increased and reached their peaks in the range of 1.3~1.4 GHz. In ventral brain regions (Pir and Amygda), BRSAR had the lowest variations (<76%) in the frequency band under observation. In other brain regions (RS, I, O, St, and Hip), the BRSAR variations first declined to valleys located in the range of 0.15~0.35 GHz and then reached two peaks. The first ones located in 0.45~0.7 GHz and the second ones located in 1.3~1.6 GHz. The highest BRSAR variations was at 1.3 GHz in RS (~191%) for PA incidence configurations. For DV incidence, the BRSAR variations nearly plateaued around 190% before 0.45 GHz and then sharply dropped to valleys in the range of 0.6~0.7 GHz (Fig. 6g–i). In most brain regions, when the frequency continued to increase, the BRSAR variations had two peaks. The first ones located at 1 GHz and the second ones located in 1.6 GHz~2 GHz. For VD incidence, the BRSAR variations nearly plateaued around 190% before 0.5 GHz and then sharply dropped to valleys in 1~2.3 GHz (Fig. 6j–l). After these valleys, the variations fluctuated ranging from 0.04% to 121.88%. For LR and RL incidence, the variations of BRSAR were similar due to the symmetric property of rats (Fig. 6m–r). The variations of BRSAR were also at plateaus around 190% before 0.45 GHz and then sharply dropped to valleys in 0.7~1.3 GHz. After the valleys, the variations fluctuated ranging from 0.03% to 128.81%.

### Reliability of using WBDSAR and WBRSAR as estimations of BRSAR

**Figure 7:**
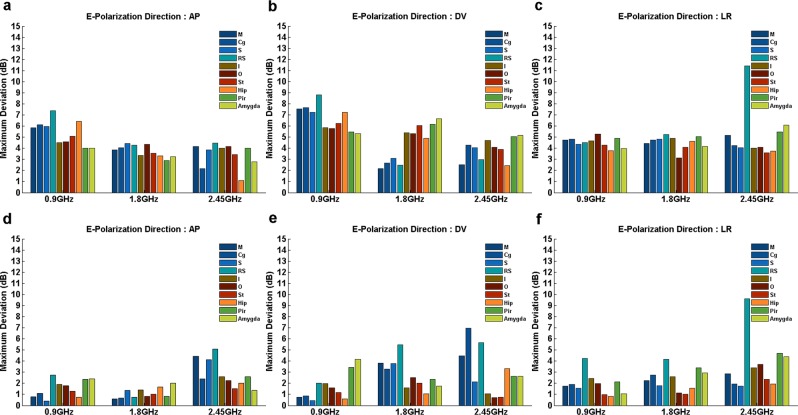
Maximum *D*_WBDSAR_ and *D*_WBRSAR_ in different brain regions at 0.9 GHz, 1.8 GHz, and 2.45 GHz for certain E-polarization directions. (**a**–**c**) Show the maximum ***D***_**WBDSAR**_ when E-polarization directions are AP, DV, and LR, respectively. (**d**–**f**) Show the maximum ***D***_**WBRSAR**_ when E-polarization directions are AP, DV, and LR, respectively. M: motor cortex; Cg: cingulate gyrus; S: sensory cortex; RS: retrosplenial cortex; I: insular cortex; O: orbital cortex; St: striatum; Hip: hippocampus; Pir: piriform cortex; Amygda: amygdaloid body.

To investigate the reliability of using WBDSAR as an estimation of BRSAR, the deviations between BRSAR and WBDSAR (*D*_WBDSAR_) were calculated at three selected frequencies (0.9 GHz, 1.8 GHz, and 2.45 GHz) at which the related bioeffects had been extensively investigated. Figure 7 shows the comparison of the maximum *D*_WBDSAR_ of certain E-polarization directions. As shown in Fig. 7a, for AP E-polarization, the Hip region had the lowest maximum *D*_WBDSAR_ at 2.45 GHz. For LR E-polarization, the RS region had an obvious high maximum *D*_WBDSAR_ (11.4 dB) at 2.45 GHz.

To investigate the reliability of using WBRSAR as an estimation of BRSAR, the deviations between BRSAR and WBRSAR (*D*_WBRSAR_) were also calculated. For AP E-polarization, the maximum *D*_WBRSAR_ was obviously smaller than the corresponding maximum *D*_WBDSAR_ at 0.9 GHz and 1.8 GHz. At 2.45 GHz, however, the maximum D_WBRSAR_ in dorsal brain regions (M, Cg, S, and RS) and Hip was slightly higher than maximum *D*_WBDSAR_. For DV E-polarization, generally speaking, the maximum *D*_WBRSAR_ was smaller than the maximum *D*_WBDSAR_ (Fig. 7e). However, at 1.8 GHz and 2.45 GHz, the maximum *D*_WBRSAR_ in the dorsal brain regions (M, Cg, and RS for both 1.8 GHz and 2.45 GHz; S for 2.45 GHz) was higher than maximum D_WBDSAR_. For LR E-polarization, all maximum *D*_WBRSAR_ were smaller than the corresponding maximum *D*_WBDSAR_ (Fig. 7f). At 2.45 GHz, the maximum *D*_WBRSAR_ in RS (9.59 dB) was much higher than the other brain regions.

## Discussion

Tremendous efforts have been made to investigate the effect of EMF radiation on the brain in past decades. The studies based on small animals, especially rats, play an important role in helping us establish a dose-effect relationship of EMF radiation and understand the mechanism behind it. Although many milestones have been achieved, accurate dosimetry analysis in sub-regions of rat brain has not been systemically investigated based on FDTD method even if the study of dose-effect relationship is based on detailed histopathology in specific brain regions (e.g. hippocampus)^17,23,24^. In this study, the influence of frequency, incidence direction, and E-polarization direction on BRSAR and the reliability of using WBDSAR/WBRSAR as estimations of BRSAR were evaluated by numerical simulations based on a voxel rat model consisting of 10 representative brain regions.

Realistic computational animal models have long been used as surrogates to characterize the anatomy structures of actual animals in EMF dosimetry analysis. Among these models, numerous voxel rat models have been proposed for specific purposes. For instance, Burkhardt *et al*.^25^ proposed a voxel rat model for dosimetry analysis in wireless communication system of 0.9 GHz. Wang *et al*.^26^ developed a pregnant rat model for dosimetry evaluation of 1.95 GHz RF radiation. Most of these voxel rat models have taken rats’ brain as a unified structure, though some researchers have considered the SAR value of white matter, grey matters, thalamus, and cerebellum, etc.^3,8,14,16^. However, none of these studies have segmented rat brains into sub-regions directly related to certain functions (e.g. hippocampus which is related to memory). The voxel rat model constructed in this study can help us to evaluate SAR distribution in rat brains at a more detailed level. It is also worth noticing that although only 10 represented brain regions have been segmented in this work, the number of brain regions can actually be up to 624^20^.

The WBDSAR and WBRSAR obtained in our work were compared with the ones reported in previous literatures^7,8^ after normalized to (W/kg)/(mW/cm^2^). The SAR values obtained in our study differed from the literature data. For instances, when the configuration of EMF was DVAP, the WBDSAR at 1.1 GHz in our study and in ref.^8^ were 0.34 (W/kg)/(mW/cm^2^) and 0.46 (W/kg)/(mW/cm^2^), respectively. The WBRSAR at 2.0 GHz in our study and ref.^7^ were 0.56 (W/kg)/(mW/cm^2^) and 1.20 (W/kg)/(mW/cm^2^), respectively. The differences of WBDSAR and WBRSAR between our work and the previous literatures may result from the dependency of SAR on the model resolution, grid resolution, and anatomic properties of the rat model such as the weight, length, specific morphological features of organs, etc.

For a certain configuration of EMF, all of the WBDSAR, WBRSAR and BRSAR depended heavily on the frequency. For WBDSAR, according to previous studies^27^, if the E-polarization direction was parallel to the axial direction of the rat, the resonance effect occurs when the ratio between the body length and the wavelength in air is about 0.4 ± 0.02. For the rat model used in this study, which was about 260 mm in length (without tail), the body resonant frequency should be around 0.45 GHz. In our study, the whole-body resonance peaks observed at 0.45 GHz with AP polarization were consistent with this prediction. For WBRSAR, Gandhi *et al*.^27^ have found that the average SAR value in the head is about 3.3 times the corresponding WBDSAR due to head resonance. Zhou *et al*.^9^ have reported additional peaks besides the whole-body resonance peaks found in human brains owing to brain resonance. Similarly, Chen *et al*.^7^ have found a second SAR peak in rat brains resulting from the joint effects induced by conductivity increasing and the skin effect at higher frequency. In our study, for AP E-polarization, there existed additional WBRSAR peaks around 1.1~1.2 GHz other than the WBRSAR peaks induced by whole-body resonance around 0.4~0.5 GHz. This result was also consistent with previous studies. The difference between the frequencies of additional WBRSAR peaks in our work and previous literature may due to different morphological characteristics in the rat models implemented in numerical simulations. For other E-polarization directions, the WBRSAR have no peaks at the whole-body resonance frequency. Instead, several WBRSAR peaks were found at higher frequencies (1.0~2.2 GHz) depending on the different configurations of EMP. For certain EMF exposure configurations, the tendencies of BRSAR along with frequency were generally similar to the corresponding WBRSAR. However, the number of SAR absorption peaks, the locations of the BRSAR peaks, and the BRSAR values at certain frequencies varies from one brain region to another. All of these knowledge depicted the dependence of SAR in rat on frequency at three different scales.

The incidence and E-polarization directions of EMF exposure could influence the SAR value of rats as well as the related biological effects. For instance, Schrot and Hawkins *et al*.^28^ demonstrated that different E-polarization directions of electromagnetic waves resulted in substantially different convulsion time for mice at identical power densities. This phenomenon may result from different SAR distributions in rats and is determined by different configurations of incidence and E-polarization directions of EMF, which depended on the setup of the EMF exposure apparatus and the posture of the rat. Chen *et al*.^7^ found that incidence and E-polarization directions can influence the WBDSAR of rats under plane wave EMF radiation. Grufstrom *et al*.^16^ reported a 56% standard deviation in their setup around WBDSAR and WBRSAR values for different rat orientations. However, the influence of incidence and E-polarization directions on BRSAR of rats has not been systemically investigated.

In this study, we found that the incidence and E-polarization directions had great impacts on BRSAR. Our results suggested that the incidence direction played an important role in BRSAR in the range of 0.6~1.7 GHz for AP E-polarization exposures. For DV E-polarization, generally speaking, the influence of incidence direction on BRSAR decreased as the frequency increased in all brain regions. For LR E-polarization, the influence of incidence direction on BRSAR maintained a relatively high level (>50%) for most brain regions at all frequencies. For some brain regions such as RS, the variation of BRSAR due to the change of incidence direction was as high as 330% at 1.5 GHz. The E-polarization direction also had significant effects on the BRSAR for all brain regions around relatively high frequencies (1.3~1.4 GHz) when the incidence direction was parallel to the axial direction of the rat (AP and PA). However, for other incidence directions (DV, VD, LR, and RL), the influence of E-polarization on BRSAR maintained a high level (~190%) at low frequencies (<0.45 GHz) for all brain regions. In summary, both incidence and E-polarization directions of EMF could influence the BRSAR in specific rat brain regions and so their specific biological functions. For the studies that aimed to establish a dose-effect relationship of EMF, either all rats should be well constrained in a certain exposure setup with identical orientation, or the BRSAR of rats should be carefully evaluated considering their movement.

Numerous studies have been conducted to investigate the biological effect on brain and its functions induced by EMF radiation^29–32^. However, most of these studies only provided WBDSAR or WBRSAR instead of BRSAR, even when the biological effects under investigation were localized in specific brain regions^12,33–35^. In this study, the deviations between BRSAR and WBDSAR/WBRSAR at three typical frequencies (0.9 GHz, 1.8 GHz, and 2.45 GHz) were investigated. At these frequencies, WBRSAR seemed to be a better estimation of BRSAR than WBDSAR for most brain regions. However, for some brain regions such as M, Cg, S, RS, and Hip, the maximum D_WBRSAR_ was larger than corresponding maximum D_WBDSAR_ for certain configurations. According to the results of our simulation, for certain configurations, the BRSAR of specific brain regions were different from both WBDSAR and WBRSAR. The largest deviation could be up to 13.1 dB and 9.59 dB across all configurations for WBDSAR and WBRSAR, respectively. Compared with the WBDSAR and WBRSAR, BRSAR is a more accurate evaluation in EMF exposure assessment because it has been calculated based on more detailed morphological features of specific brain regions. The use of WBDSAR and WBRSAR without carefully consideration may lead to an inaccurate dose-effect relationship especially for the studies focused on specific brain regions.

There are still several deficits in this work. First, the effect induced by multiple rats has not been considered. Under plane wave EMF radiation, for rats that are close to one another, the SAR value will be different from the isolated rat in free space^27^. Thus, the modification of SAR distribution induced by multiple rats should be further investigated. Second, in this study, only the case of plane wave EMF exposure was considered. For other setups of EMF exposure such as dipole antenna^25,36,37^, Transverse Electromagnetic (TEM) cell^16,32,38^, circular wave guide^24,39^, and other specifically designed local exposure systems^40^, the SAR distribution in different brain regions should also be evaluated carefully. Third, the resolution of rat model also have influence on the estimation of SAR^21^. With a better model resolution, more accurate estimation of BRSAR as well as the variances induced by the directions of EMF exposure could be obtained.

In conclusion, the BRSAR values in different brain regions of rats depend on the frequency, incidence direction, and E-polarization direction of EMF exposure. To establish an accurate dose-effect relationship, the variance of BRSAR value induced by the alteration of these parameters should be avoided or carefully evaluated. Furthermore, the use of WBDSAR and WBRSAR as estimations of BRSAR should be restrained to certain conditions such that the deviations are not too large. This work could help in improving the designs of future studies on dose-effect relationship of EMF exposure in order to guarantee their reliability.

## Supplementary information

Supplementary Information

## Data Availability

All data generated or analysed during this study are included in this published article (and its Supplementary Information File).
