# Supplementary material for: The specific absorption rate in different brain regions of rats exposed to electromagnetic plane waves
Source: Sci Rep. 2019 Sep 16;9:13277. doi: 10.1038/s41598-019-49719-4 (PMC6746715; doi:10.1038/s41598-019-49719-4)
Supplement: Supplementary file 1 — Supplementary Information [file 41598_2019_49719_MOESM1_ESM.docx]

**The specific absorption rate in different brain regions of rats exposed**

**to** **electromagnetic plane waves**

Hao-Yu Wang^1†^, Chun-Fang Li^1,2†^, Chao Yu^1†^, Ji Dong^1^, Yong Zou^1^, Bin-Bin Nie^3^,

Jia-Kai Li^4^, Lin Ma^2^, Rui-Yun Peng^1*^

^1^ Beijing Institute of Radiation Medicine, Beijing, China

^2^ First Medical Center of PLA General Hospital, Beijing, China

^3^ Institute of High Energy Physics, Chinese Academy of Sciences, Beijing, China

^4^ Hainan Hospital of PLA General Hospital, Sanya, Hainan, China

^†^ These authors contribute equally to this work

^*^ Corresponding author

**Supplementary Figures**

**
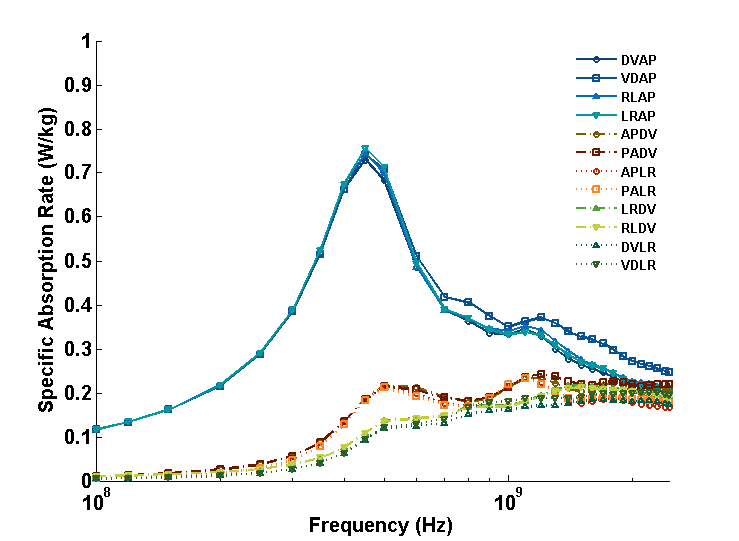
**

**Supplementary Fig. S1 WBDSAR of rat vs frequency for different EMF exposure configurations.** For the definitions of DVAP, VDAP, RLAP, LRAP, APDV, PADV, APLR, PALR, LRDV, RLDV, DVLR, and VDLR, see Table 1 in the main article.

**
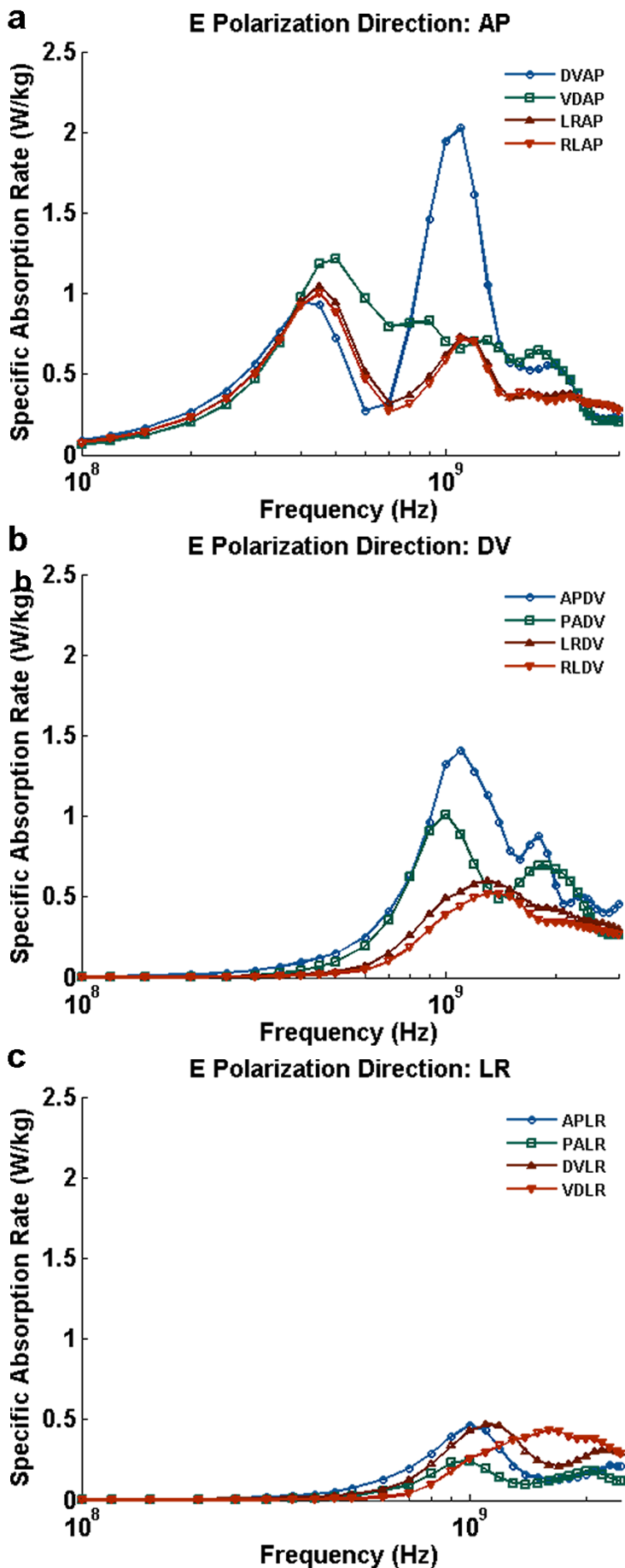
**

**Supplementary Fig. S2 WBRSAR of rat vs frequency for different EMF exposure configurations.** a, b, and c illustrate the WBRSAR at different frequencies in semilog coordinates when the E-polarization directions are AP, DV, and LR, respectively. For the definitions of DVAP, VDAP, RLAP, LRAP, APDV, PADV, APLR, PALR, LRDV, RLDV, DVLR, and VDLR, see Table 1 in the main article.

**Supplementary Tables**

**Supplementary Table 1S．**Properties of brain sub-regions.

| **Brain Regions** | **Weight (mg)** | **Volume (mm^3^)** |
| --- | --- | --- |
| M | 86.55 | 82.78 |
| Cg | 32.58 | 31.16 |
| S | 196.10 | 187.50 |
| RS | 51.09 | 48.87 |
| I | 45.25 | 43.28 |
| O | 30.61 | 29.28 |
| St | 121.70 | 116.40 |
| Hip | 156.70 | 149.90 |
| Pir | 63.94 | 61.16 |
| Amygda | 55.24 | 52.84 |

**Supplementary Table 2S．**Relative permittivity *ε*_r_ and conductivity *σ* of brain at different frequencies ranging from 0.1GHz to 2.45GHz.

| **Frequency (GHz)** | ***ε*_r_** | ***σ* (S/m)** |
| --- | --- | --- |
| 0.10 | 89.7700 | 0.7903 |
| 0.12 | 82.1089 | 0.8192 |
| 0.15 | 74.5592 | 0.8547 |
| 0.20 | 67.1118 | 0.9018 |
| 0.25 | 62.6762 | 0.9401 |
| 0.30 | 59.7194 | 0.9732 |
| 0.35 | 57.5983 | 1.0030 |
| 0.40 | 55.9962 | 1.0304 |
| 0.45 | 54.7387 | 1.0562 |
| 0.50 | 53.7221 | 1.0808 |
| 0.60 | 52.1703 | 1.1279 |
| 0.70 | 51.0315 | 1.1733 |
| 0.80 | 50.1514 | 1.2181 |
| 0.90 | 49.4442 | 1.2628 |
| 1.00 | 48.8582 | 1.3081 |
| 1.10 | 48.3607 | 1.3540 |
| 1.20 | 47.9294 | 1.4009 |
| 1.30 | 47.5492 | 1.4490 |
| 1.40 | 47.2091 | 1.4982 |
| 1.50 | 46.9008 | 1.5488 |
| 1.60 | 46.6185 | 1.6008 |
| 1.70 | 46.3574 | 1.6541 |
| 1.80 | 46.1138 | 1.7089 |
| 1.90 | 45.8849 | 1.7652 |
| 2.00 | 45.6684 | 1.8230 |
| 2.10 | 45.4623 | 1.8823 |
| 2.20 | 45.2653 | 1.9430 |
| 2.30 | 45.0760 | 2.0053 |
| 2.40 | 44.8933 | 2.0691 |
| 2.45 | 44.8041 | 2.1015 |
